# Spatial Determinants of Ebola Virus Disease Risk for the West African Epidemic

**DOI:** 10.1371/currents.outbreaks.b494f2c6a396c72ec24cb4142765bb95

**Published:** 2017-03-31

**Authors:** Kate Zinszer, Kathryn Morrison, Aman Verma, John S. Brownstein

**Affiliations:** School of Public Health and Public Health Research Institute, University of Montreal, Montreal, Quebec, Canada; School of Public Health and Public Health Research Institute, University of Montreal, Montreal, Quebec, Canada; Clinical and Health Informatics, McGill University, Montreal, Quebec, CanadaMcGill University; Department of Pediatrics, Harvard Medical School and Children’s Hospital Informatics Program, Boston Children’s Hospital, Boston, Massachusetts, United States of America

**Keywords:** ebola, Guinea, Liberia, Sierra Leone, spatial analysis, statistical model

## Abstract

Introduction: Although many studies have investigated the probability of Ebola virus disease (EVD) outbreaks while other studies have simulated the size and speed of EVD outbreaks, few have investigated the environmental and population-level predictors of Ebola transmission once an outbreak is underway. Identifying strong predictors of transmission could help guide and target limited public health resources during an EVD outbreak. We examined several environmental and population-level demographic predictors of EVD risk from the West African epidemic. Methods: We obtained district-level estimates from the World Health Organization EVD case data, demographic indicators obtained from the Demographic and Health surveys, and satellite-derived temperature, rainfall, and land cover estimates. A Bayesian hierarchical Poisson model was used to estimate EVD risk and to evaluate the spatial variability explained by the selected predictors. Results: We found that districts had greater risk of EVD with increasing proportion of households not possessing a radio (RR 2.79, 0.90-8.78; RR 4.23, 1.16-15.93), increasing rainfall (RR 2.18; 0.66-7.20; 5.34, 1.20-23.90), and urban land cover (RR 4.87, 1.56-15.40; RR 5.74, 1.68-19.67). Discussion: The finding of radio ownership and reduced EVD transmission risk suggests that the use of radio messaging for control and prevention purposes may have been crucial in reducing the EVD transmission risk in certain districts, although this association requires further study. Future research should examine the etiologic relationships between the identified risk factors and human-to-human transmission of EVD with a focus on factors related to population mobility and healthcare accessibility, which are critical features of epidemic propagation and control.

## INTRODUCTION

The recent Ebola virus disease (EVD) epidemic in West Africa was the largest EVD outbreak in history, spreading across Guinea, Liberia, and Sierra Leone, infecting an estimated 28,600 individuals, and claiming over 11,000 lives.^1^ Numerous factors contributed to the human-to-human spread of EVD, including individual-level factors such as caring for infected individuals and involvement in funeral preparations of infected corpses, as well as systemic and upstream factors such as inadequate healthcare infrastructure.^2^ Mobile populations coupled with porous borders^3^ and commercial air travel^4^ resulted in EVD transmission outside of the epicenter of the outbreak to neighbouring countries including Nigeria, Mali, Senegal, and to other continents including North America and Europe.^5^

EVD has caused numerous outbreaks, the majority in equatorial Africa, since the first human outbreaks were detected in 1976 in the Democratic Republic of Congo and South Sudan.^6^ Five species of Ebolavirus have been isolated: Bundibugyo, Côte d’Ivoire (Taï Forest), Reston, Sudan, and Zaire.^7^ Bundibugyo, Sudan, and Zaire are responsible for the majority of human-related outbreaks with the strain in the West African epidemic belonging to the Zaire species. Fruit bats are believed to be a key reservoir of Ebolavirus, which can also cause illness and death in non-human primates such as in apes and monkeys.^8^ Human outbreaks are typically started when an individual has come into contact with the blood of an infected mammal or bushmeat6 although the West African outbreak is thought to have begun because of fruit bat exposure.^9^ Human-to-human transmission is then propagated through direct contact with infected individuals and cadavers.^10^ Illness in humans appears two to 21 days after infection and the initial symptoms are fever, headache, and myalgia. These symptoms are followed by vomiting, diarrhea, rash, impaired liver and kidney functions, and can also result in internal and external bleeding.^8^ The case fatality rate for the most recent outbreak was estimated at 71% during the first nine months of the epidemic, which is similar to other outbreaks of Zaire ebolavirus species (60%-90%) and higher than outbreaks from Sudan ebolavirus species (40%-60%).^11^

Several studies have identified predictors of Ebola ecological niches (occurrence of environmental conditions that support its presence in a particular location),^12^ spillover events (introduction into human populations), or the onset of EVD outbreaks. The identified predictors have included extensive EVD-related deaths in primates;^13^ deforestation and human forest activities;^14^ population density;^15^ elevated levels of precipitation,^16^ humidity,^17^ and elevation;^12^ the transition from rainy to dry season;^14^^,^^18^ moderate-to-high temperatures^16^ and lower temperatures in equatorial Africa;^12^^,^^17^ as well as increased vegetation density^12^ and evergreen broadleaf forest coverage^16^.

Previous work has investigated the probability of Ebola outbreaks, and mathematical modelling studies have estimated the size, speed, and spatio-temporal patterns of EVD using simulated data.^9^^,^^10^^,^^12^^,^^13^^,^^16^^,^^17^^,^^19^^,^^20^^,^^21^^,^^22 ^However, further evidence is needed to guide public health control efforts during an outbreak, and empirical analysis of real data from the West African epidemic may yield important insight into the successful control of future outbreaks. There have been limited studies that have assessed the spatial distribution of EVD human cases using empirical data; Stanturf et al.^23^ found that social vulnerability was qualitatively and positively associated with the spatial EVD transmission in Liberia and Rainisch et al.^24^ found that spatial risk of EVD infection was as related to population, cases, and distance between affected and unaffected areas. In this study, we sought to identify environmental and population-level demographic spatial predictors of human EVD risk from the recent West African epidemic.

**MATERIALS AND METHODS**

**Study area**

The study included the three West African countries with widespread EVD transmission: Guinea, Liberia, and Sierra Leone. Guinea has an estimated population of 11,780,000^25^ and covers an area of 245,860 km^2^ including terrain consisting of costal mangrove plains, forested highlands, and savannah plains.^26^ Guinea has a tropical climate with a rainy season (April to October) and a dry season (November to March).^25^ Liberia has an area of 111,370 km^2^ with an estimated population of 4,196,000. Its terrain ranges from sandy coastal plains to rolling hills and rolling plateau, with low mountains in the northeast.^26^ Liberia has a warm, humid climate with a rainy season from May to October and a dry season from November to April. Sierra Leone’s estimated 5,879,000 population is contained within an area of 71,740 km.^25^ It has coastal mangrove swamps, wooded hills, an upland plateau and mountains in the east. Sierra Leone experiences a tropical climate with a rainy season from May to October and a dry season from November to April.^26^

**Data**

Publicly available data from the World Health Organization (WHO) included weekly counts of confirmed EVD by prefecture (Guinea, n=34), county (Liberia, n=15), and district (Sierra Leone, n=14) as of May 13, 2015.^27 ^We calculated the cumulative confirmed cases from these data, which was our outcome of interest. We then explored correlations between various environmental and socio-demographic variables, as a first step in identifying predictors to include in our final model.

Data for potential predictors were obtained from multiple sources including satellite sensor-derived environmental data and national Demographic and Health Surveys (DHS). Rainfall, temperature, and land cover measures were obtained from the Tropical Rainfall Measuring Mission (TRMM) and moderate resolution imaging spectroradiometer (MODIS) instruments onboard the Terra satellite. The TRMM product (TRMM3B42RT) provided weekly accumulated rainfall estimates with a spatial resolution of 0.25° × 0.25°, which was then weighted by the surface area daytime and nighttime land surface temperature (LST) estimates were obtained from MODIS (MOD11A2) using eight-day composite images at a 1 km × 1 km resolution. TRMM and LST estimates were obtained between April 28, 2014 and May 3, 2015 and 17 land cover classifications were provided by MODIS (MCD12Q1) at a 500 m × 500 m resolution for the most recent year available (2012). Eight of the land cover classifications were considered in the analysis as the remaining nine categories had negligible presence in Guinea, Liberia, and Sierra Leone. Density of waterways and roadways were estimated (km per km^2^), as was the average elevation and land area from shapefiles obtained from DIVA-GIS. Shapefiles from the WorldPop project were acquired, which provided projected population estimates. All estimates were obtained at the district level and Universal Transverse Mercator zone 28 projection was used.

National DHS from Guinea (2012), Liberia (2013), and Sierra Leone (2013) were used for subnational estimates on household education, wealth, occupation, household structure, and possessions and amenities. For Liberia and Sierra Leone, these estimates were obtained for the county and district levels respectively. For Guinea, DHS estimates were only available at the regional level (n=8), therefore prefectures within each region were given the same DHS-derived values.

**Covariate selection**

Linearity between covariates and the outcome was first assessed and for ease of interpretation and to avoid modelling complex non-linear terms, all continuous variables were reclassified into tercile intervals. Simple transformations (e.g., exponential, log, quadratic) were insufficient to produce a linear relationship between the covariate and outcome. Multicollinearity between ternary covariates was then examined using Cramer’s V^28^ and if two or more variables had correlations under 0.4, we fit one bivariate Poisson regression model for each variable with the outcome, and selected the covariate whose model had the lowest Akaike information criterion (AIC).

We regressed the cumulative total of EVD cases (outcome) on the selected covariates using a Poisson model with the total population per district as an offset, given the population differences between the districts. We used the glmulti^29^ R package to exhaustively explore the covariate subset and selected the covariate subset whose model had the best AIC.^30^

**Assessment of spatial variance**

Having identified a covariate subset, we quantified the amount of variation in EVD cases that this covariate set explained by using a spatial autoregressive modelling approach.^31^ We expanded our multivariable Poisson model to include two random effects: one explaining uncorrelated residual variation (u), and one explaining spatially correlated residual variation (ν).^32^ Although this approach to modelling disease variability across space has been widely used in the spatial epidemiologic literature,^33^^,^^34^^,^^35^ we use this approach to qualitatively assess the spatial and non-spatial variation explained by covariate subset. The model is as follows:

\begin{equation*}Y_i \sim Poisson(\mu_i) \\\%0Alog(\frac{Y_i}{pop_i}) = \alpha_0 + \beta_1x_1 + \beta_2x_2 + ... + \beta_{12}x_{12} + u_i + \nu_i \\\%0A\end{equation*}

\begin{equation*}u_i \sim N(0, \frac{1}{\tau_u})   \\\%0A\nu_i | \tau_\nu, \nu_j, i \neq j, \sim N(\frac{1}{n_i} \sum_{i\sim j} \nu_j, \frac{1}{n_i\tau_\nu}) \\\%0A\alpha_0, \beta_1, \dots, \beta_j \sim N(0, 1000)\end{equation*}

where *i* and *j* refer to two distinct regions in the study area, *i* ~ *j* refers to two neighboring regions, and *n_i_* refers to the number of neighboring regions for region *i*. The covariates from x_1_ to x_1__2 _are defined in Table 1. T2 and T3 refer to the second and third terciles. Non-informative priors were used for the variance and the regression coefficients, and sensitivity analyses suggested that the priors did not significantly affect the marginal posteriors of the parameters of interest.

**Table 1 Covariates:** T2=second tercile, T3=third terciles; *Kilometers of roadway per 100 km^2^ of land area; †Number of people per km^2^ of land area; ‡Proportion (%) of total land surface area; §Households without radio possession; ¶Mean years of education of head of household.

Parameter	Parameter value
x_1_	Rainfall T2
x_2_	Rainfall T3
x_3_	Roadway T2*
x_4_	Roadway T3
x_5_	Population T2†
x_6_	Population T3
x_7_	Urban T2‡
x_8_	Urban T3
x_9_	Radio T2§
x_10_	Radio T3
x_11_	Education T2¶
x_12_	Education T3

Spatial hierarchical models were fit using Bayesian estimation via the R-INLA package.^36^ R-INLA uses integrated nested Laplace approximations to estimate marginal posterior distributions for each parameter, rather than a simulation-based approach like Markov chain Monte Carlo (MCMC) methods.^37^ Approximations using INLA have been shown to be extremely accurate based on simulation studies and comparisons to well-performing MCMC, and are more computationally efficient than standard MCMC software implementation options such as WinBUGS.^38^ Two spatial models were fit using R-INLA; the first was a null model including only the outcome (EVD cases) with a population offset, an intercept, and the two random effects (uncorrelated variation and spatially correlated variation). For the second (full) model all of the selected covariates were added to the null model. This allowed for the visual assessment of areal-level spatial clustering of EVD cases that was explained by the selected covariates.

The analysis was conducted in R version 3.2.1 software and STATcompiler was used for DHS indicators included in Table 1, to obtain country-level values.

**RESULTS**

Table 2 summarizes selected covariates at the country-level for Guinea, Liberia, and Sierra Leone. Sierra Leone had the largest number of confirmed cases as well as the highest road and waterway density. Liberia and Guinea were similar in case burden, with Liberia having the largest portion of households headed by females and households without toilets. Guinea had the highest elevation as well as the highest proportion of households with electricity.

**Table 2 Country-level summaries for selected covariates using mean values:** *Total confirmed EVD cases as of May 13, 2015; †Kilometer of roadway or waterway per 100 km^2^ of land area; ‡Proportion (%) of total land surface area; §Proportion (%) of households headed by males who have completed secondary education; ¶Proportion (%) of households that drink surface water source such as river, canal, dam, irrigation channel, lake, pond, and stream.

Covariate	Guinea	Liberia	Sierra Leone
Total confirmed EVD cases*	3,144	3,339	9,394
Average rainfall accumulation (cm)	3.2	3.6	4.8
Average elevation (m)	3.2	172.1	166.4
Roadway density (km)†	10.0	11.2	16.8
Waterway density (km)†	11.2	9.0	15.7
Cropland (%)‡	9.5%	5.1%	13.3%
Female headed households (%)	17.3%	35.2%	28.0%
Secondary education (%)§	1.5%	10.5%	4.8%
Households (%) without toilets	19.5%	45.2%	21.4%
Households (%) with drinking water	10.2%	15.4%	18.2%
Households (%) with electricity	26.2%	9.8%	13.5%
Households (%) with radios	61.5%	58.9%	58.8%

Figure 1 displays the correlation between the covariates that were included in a final model, which was less that 0.4 for all.

**Figure:**
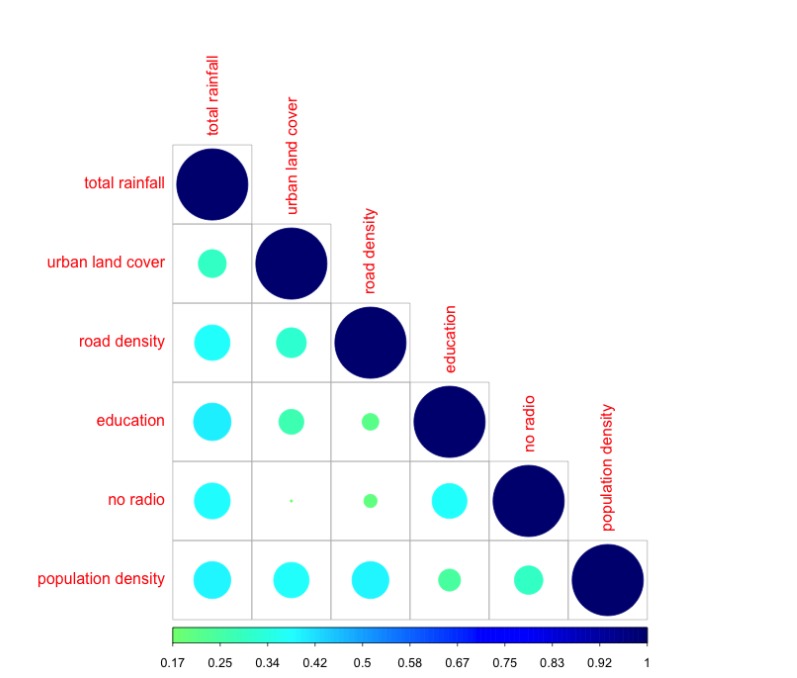
Figure 1: The color and circle size vary with correlation, with increasing circle size and intensity in the color blue represent increasing correlation.

Table 3 presents the median posterior rate ratios for the covariates that were included in the final model. We report medians of the marginal posterior distributions for each parameter as our point estimates, as commonly done in Bayesian analyses. The spatial analysis indicates that districts were more at risk of EVD with increasing rainfall (RR_Rainfall2_ 2.18; 95% credible interval 0.66-7.20; RR_Rainfall3_ 5.34, 1.20-23.90), urban land cover (RR_Urban2_ 4.87, 1.56-15.40; RR_Urban3_ 5.74, 1.68-19.67), households not possessing a radio (RR_Radio2_ 2.79, 0.90-8.78; RR_Radio3_ 4.23, 1.16-15.93), and years of education (RR_Education3_ 1.58, 0.40-6.25). Districts with low density were at higher risk than those with medium population density (RR_Population2_ 0.64, 0.18-2.32) and similarly, districts with low or high roadway density (RR_Roadway3_ 1.22, 0.35-4.26) were at higher risk those with medium roadway density (RR_Roadway2_ 0.61, 0.19-1.96).

**Table 3 Rate ratio posterior median estimates from full multivariable spatial model:** *Kilometers of roadway per 100 km^2^ of land area; †Number of people per km^2^ of land area; ‡Proportion (%) of total land surface area. §Mean years of education of head of household.

Covariate	Rate rate (95% credible interval)
Weekly rainfall accumulation (cm)	
<3.2	1.00
3.2-4.2	2.18 (0.66, 7.20)
>4.2	5.34 (1.20, 23.90)
Roadway density*	
<0.09	1.00
0.09-0.11	0.61 (0.19, 1.96)
>0.11	1.22 (0.35, 4.26)
Population density†	
<33.6	1.00
33.6-68.0	0.64 (0.18, 2.23)
>68.0	0.98 (0.22, 4.35)
Urban land cover (%)‡	
<0.02	1.00
0.02-0.09	4.87 (1.56, 15.40)
>0.09	5.74 (1.68, 19.67)
Household not possessing radios (%)	
<38.1	1.00
38.1-47.6	2.79 (0.90, 8.78)
>47.6	4.23 (1.16, 15.93)
Years of education§	
<2.1	1.00
2.1-3.2	0.98 (0.25, 3.79)
>3.2	1.58 (0.40, 6.25)

In the full model, the spatial residuals are very small in magnitude and appear to be spatially random whereas the uncorrelated non-spatial residuals suggest that unmeasured non-clustered variation remains. In other words, the covariate set explains a meaningful amount of spatial variation but there are additional unmeasured or unmeasurable factors that explain the different rates of EVD per district.

**DISCUSSION**

In this study, we identified several environmental and demographic spatial predictors of EVD risk at the district level for Guinea, Liberia, and Sierra Leone, which require further study to determine causality. We found that lack of radio ownership was a strong predictor of EVD risk (RR_Radio2_ 2.79, 0.90-8.78; RR_Radio3_ 4.23, 1.16-15.93) at the district level. Radio campaigns in all three countries used serial dramas and popular music to disseminate risk communication, prevention, and social mobilization messages, which may have reduced EVD transmission risk.^39^^,^^40^^,^^41^ Future work should further examine this association and also consider cost-effectiveness analysis of alternative methods of health message dissemination for places where household radio ownership is low.

The correlation between rainfall and EVD transmission risk is supported by previous work which found associations between increased rainfall or humidity and EVD outbreaks.^16^^,^^17^ Roads can become impassable with higher levels of rainfall, which may make it more difficult to seek healthcare treatment,^23^ and to implement infection control measures, increasing EVD transmission risk. A time series approach to examining this relationship may provide further insight into the association between rainfall and EVD, coupled with improved road accessibility and health facility data.

We included roadway and waterway densities as proxies for population mobility, which is thought to have been an important influence in the explosive nature of West African EVD epidemic.^12^^,^^42^^,^^43^ Roadway density had a U-shaped association with EVD risk with the second tercile being protective against EVD risk and the third tercile having a slightly increased risk of EVD. Higher roadway density could decrease risk by improving accessibility to treatment centers, but this association could also be an artifact of measurement error, as the roadway data was from 2007.

Surprisingly, population density had no association with EVD risk with the exception of the second tercile having a weak protective effect (RR_Population2_ 0.64, 0.18-2.32). This suggests that lower population densities were at increased risk for EVD transmission, which could be a consequence of clinical and public health service provision issues in remote areas. Conversely, our finding of increased EVD risk in more urban areas (RR_Urban2_ 4.87, 1.56-15.40; RR_Urban3_ 5.74, 1.68-19.67), while controlling for population density, may reflect the population mobility and the increased mixing between susceptible and infected individuals in urban areas.

Previous EVD outbreaks had much fewer cases and differences have been noted in historical outbreaks when EVD was introduced into the general population versus into a healthcare setting.^44^ General population outbreaks were small and appear to end spontaneously with limited generations of cases, whereas healthcare settings with low standards of hygiene and sanitation can amplify transmission and result in a high number of cases and deaths of healthcare workers.^44^^,^^45^ The West African EVD epidemic was a mixture of nosocomial and general population settings, which sustained human-to-human transmission due to various reasons including burial practices, inadequate infection control, population density and mobility, cultural beliefs and practices, and fear.^2^^,^^23^^,^^42^ We were not able to measure several of the risk factors for human-to-human transmission given a lack of sufficient data at the district level and examination of the residuals supports the need to include other factors in the model.

There are different ways in which measurement error could have influenced our findings. Remote sensing data was used in lieu of ground observations due to data availability and deriving measures of environmental characteristics from remotely sensed data requires assumptions about the values, which are also subject to measurement error.^46^^,^^47^^,^^48^ We did not have DHS estimates for each prefecture in Guinea, therefore coarser region-level estimates were used, which may have biased our effect estimates toward the null. Additionally, the DHS data were captured from different time periods than the remote sensing and WHO data and consequently, the district-level values for the indicator may have changed from the date of data collection to the EVD epidemic. We use ecological (aggregate) data to determine district-level predictors of EVD risk, which can lead to biases if used to interpret individual-level associations for individuals within districts between EVD risk and predictors of EVD infection.^49^^,^^50^

Our work has shed new light on population-level spatial factors for EVD risk and future research should examine the etiologic relationships of these risk factors and EVD transmission. The potentially significant role of radio having reduced the EVD risk requires further study and is an important and modifiable risk factor for future outbreaks. Future research should incorporate higher spatial resolution (e.g., sub-prefectures, districts, chiefdoms) and a temporal dimension, as it would provide further understanding into aspects of population mobility and healthcare accessibility, which are critical features of epidemic propagation and control. In addition, these findings should be compared to other diseases that are transmitted from human-to-human in Guinea, Liberia, and Sierra Leone. This would provide further information into disease transmission patterns in Guinea, Liberia, and Sierra Leone and common risk factors among different diseases that could be used for integrated outbreak management.

## Competing Interests

The authors have declared that no competing interests exist.

## Data Availability

The World Health Organization’s Ebola data is publicly available from the Ebola data and statistics page (http://apps.who.int/gho/data/node.ebola-sitrep.quick-downloads?lang=en). The Demographic and Health Survey (DHS) data is publicly available for registered users from the DHS Program (http://dhsprogram.com/data/available-datasets.cfm). Satellite images were provided by NASA for rainfall estimates (https://pmm.nasa.gov/data-access/downloads/trmm) and by USGS for temperature (https://lpdaac.usgs.gov/dataset_discovery/modis/modis_products_table/mod11a2) and land cover (https://lpdaac.usgs.gov/dataset_discovery/modis/modis_products_table/mcd12q1).

## Corresponding Author

Kate Zinszer (kate.zinszer@umontreal.ca)
